# Predation impacts brain allometry in female guppies (*Poecilia reticulata*)

**DOI:** 10.1007/s10682-022-10191-8

**Published:** 2022-06-21

**Authors:** Regina Vega-Trejo, Catarina Vila-Pouca, David J Mitchell, Alexander Kotrschal

**Affiliations:** 1 Department of Zoology, Stockholm University, Svante Arrhenius väg 18B, 10691 Stockholm, Sweden; 2 Department of Zoology, Edward Grey Institute, University of Oxford, OX1 3SZ Oxford, UK; 3 Department of Animal Sciences: Behavioural Ecology, Wageningen University & Research, 6708 Wageningen, WD Netherlands

**Keywords:** Survival, Guppy, Natural selection, Phenotypic plasticity, Brain size evolution

## Abstract

Cognitive and sensory abilities are vital in affecting survival under predation risk, leading to selection on brain anatomy. However, how exactly predation and brain evolution are linked has not yet been resolved, as current empirical evidence is inconclusive. This may be due to predation pressure having different effects across life stages and/or due to confounding factors in ecological comparisons of predation pressure. Here, we used adult guppies (*Poecilia reticulata*) to experimentally test how direct predation during adulthood would impact the relative brain size and brain anatomy of surviving individuals to examine if predators selectively remove individuals with specific brain morphology. To this end, we compared fish surviving predation to control fish, which were exposed to visual and olfactory predator cues but could not be predated on. We found that predation impacted the relative size of female brains. However, this effect was dependent on body size, as larger female survivors showed relatively larger brains, while smaller survivors showed relatively smaller brains when compared to control females. We found no differences in male relative brain size between survivors and controls, nor for any specific relative brain region sizes for either sex. Our results corroborate the important, yet complex, role of predation as an important driver of variation in brain size.

## Introduction

Predation pressure is a key ecological factor in shaping the evolution of morphological, physiological, behavioural, and life-history traits (Reznick and Endler 1982; Lima and Dill 1990; Heinen-Kay and Langerhans 2013). One key trait for which predation has been identified as an important evolutionary selective force is vertebrate brain size (Burns and Rodd 2008; Kotrschal et al. 2015; van der Bijl et al. 2015; Walsh et al. 2016). This stems from the hypothesis that individuals may differ in their ability to assess predators due to differences in cognitive abilities associated with both absolute and relative brain size (Striedter 2005; Moller and Erritzoe 2014; Samuk et al. 2018). Larger-brained prey should be more effective at avoiding predators given their likely better ability to alter their behavioural responses to specific predator encounters (Shultz and Dunbar 2006). Furthermore, predicting the likelihood of a predator attack requires gathering and processing information, at which individuals with relatively larger brains for their body size might be better (Moller and Erritzoe 2014; van der Bijl et al. 2015). Indeed, this idea has been supported in several experimental and observational studies. For instance, in guppies artificially selected for large and small relative brain size (i.e. brain size relative to body size), large-brained females survived longer under predation in a semi-natural setting (Kotrschal et al. 2015) and large-brained females also spent less time inspecting a predator, indicating better risk assessment skills (van der Bijl et al. 2015). Predator-prey comparisons across different fish species have also revealed that predatory species with larger relative brain size tend to prey on larger-brained species, but that the relative brain size of the prey is typically larger than that of the predator (Kondoh 2010). This may be due to an ‘arms-race’ between predators and prey, associated with the cognitive skills required from predators to learn to find or handle prey, and from prey to learn how to avoid being eaten (Kondoh 2010).

Despite some studies supporting the idea that predation may select for a larger brain size via its contribution to cognitive abilities, the relationship between predation and brain morphology remains unresolved, with ecological comparisons and experimental work showing effects varying in magnitude, direction, heritability, and with sex (Gonda et al. 2012, 2013; Kotrschal et al. 2015; Walsh et al. 2016; Samuk et al. 2018). Although large brains may be better at processing key stimuli to detect and avoid predators, it is possible that having a larger brain can be detrimental in certain predatory environments, if its high metabolic costs outweigh the cognitive benefits (Niven and Laughlin 2008). Some studies indeed indicate that predation may also favour smaller relative brain sizes. For example, male adult Trinidadian killifish (*Rivulus hartii*) have evolved smaller brains relative to body weight in sites with predators (Walsh et al. 2016). Further, greater environmental complexity linked to predators or exposure to predators in a mesocosm setting has been associated with individuals with smaller relative brain sizes in sticklebacks in wild (Gonda et al. 2011) and laboratory experiments (Samuk et al. 2018), respectively. Yet, no link between predation and adult relative brain size was found in Trinidadian killifish when comparing sites that differ in juvenile predation (Beston et al. 2017), highlighting the need to account for the life history stage at which individuals experience predation and the type of predator.

One cause for the ambiguous results on the relationship between predation and the brain may be the role of additional, unaccounted environmental factors. For instance, predation also drives population demographics by reducing densities and changing inter and intraspecific competition dynamics (Magurran and Phillip 2001; Reznick et al. 2001, 2012). The brain is the most energetically expensive tissue, meaning factors such as population density and competition which affect resource availability are also likely to affect brain size through energetic trade-offs (Isler and van Schaik 2006), and may thus provide an explanation for the contrasting results between brain size and predation regimes of several studies.

Understanding the effect of predation on brain size variation is further complicated by the fact that predators may not only drive prey trait evolution over generations through non-random mortality (natural selection), but also by eliciting changes within a generation (phenotypic plasticity; Lima and Dill 1990; Kondoh 2010). Indeed, several behavioural and morphological traits show pronounced plastic responses to predation (Miner et al. 2005; Callahan et al. 2008), including brain size (Reddon et al. 2018). For example, teleost fish show neurogenesis during adulthood (Zupanc and Sîrbulescu 2011), enabling phenotypic changes in brain morphology in response to their environment (Ebbesson and Braithwaite 2012).

Without additional work, ecological comparisons of brain size in wild individuals from different predation regimes therefore cannot disentangle the effect of direct selection by predators from plasticity-driven variation, and these two mechanisms together may contribute to the inconsistent results reported to date. For example, Reddon et al. (2018) found that male guppies from wild high-predation populations showed relatively heavier brains than their low-predation counterparts. Yet, male guppies exposed to predation cues in the laboratory during development also had heavier brains than males exposed to control cues, indicating that some of the differences in brain size investment are at least partially due to phenotypic plasticity (Reddon et al. 2018).

In summary, despite a wealth of studies showing correlations between predation and several aspects of the brain (in terms of size and of anatomy of different regions), the link between predation and brain morphology remains ambiguous, as are the relative roles of selection by predators and plastic changes induced by predation. Importantly, it remains untested if predators can indeed exert a direct effect on the brain by selectively removing prey with specific brain morphology.

In this study, we examined how direct predation impacts brain allometry and the relative size of different brain regions in the guppy (*Poecilia reticulata*), while controlling for plastic effects of visual and olfactory predation cues. All individuals were bred and raised in similar conditions and were sexually mature young adults when exposed to a predator for the first time. We also controlled for potential foraging effects by providing food ‘*ad libitum*’. As such, we could empirically test the effect of direct removal by predators on brain size and brain anatomy in the adult stage.

We predicted that fish surviving a predation event would have a larger relative brain size, and more specifically larger structures related to perception or learning (in particular telencephalon and optic tectum), than fish from the control treatment. This is because the telencephalon is associated with certain cognitive skills that may help escape predators, such as spatial learning and memory and inhibitory control in fish (Broglio et al. 2003; Triki et al. 2021), which could increase the accuracy or speed of decisions. Similarly, electrical stimulation in the optic tectum elicits coordinated body movements and motor patterns (Broglio et al. 2003), that would allow individuals to have a better response to avoid predators. Indeed, both brain regions have been shown to be positively associated with predator pressure in the wild in a correlational population comparison (Kotrschal et al. 2017).

## Methods

We examined the effect of direct predation on relative brain size by comparing fish that were exposed to visual and olfactory cues of a predator (*Crenicichla alta*) but could not be predated (control treatment) with fish that survived exposure to a predator (predation treatment). Experiments were conducted at Stockholm University, Sweden from November 2019 to December 2020.

We used laboratory descendants of Trinidadian guppies originating from large high predation populations, but that have not been exposed to predators for 14 years. These fish are laboratory descendants of fish from a high predation population from the Quare River, Trinidad, collected in 2005. Stock populations had been kept in several large tanks, without predators, at Trondheim University. In 2010, 150 animals were brought to Sweden to start a stock population (Kotrschal et al.^2013^). In 2018, we brought more animals from Trondheim to Stockholm to set up 110 breeding pairs, from which individuals for three replicates were produced. We additionally included 100 juveniles from the primary Stockholm stock population.

Juveniles were kept in 4 L tanks until their sex could be identified (females by their gravid spot, and males by the presence of a modified anal fin called a gonopodium). Mature individuals were kept in single-sex 50 L tanks until the start of the experiment. For each replicate, a total of 200 mature individuals of each sex were used; 180 individuals were randomly selected for the predation treatment and 32 individuals for the control treatment, and we conducted three replicates per sex.

**Fig. 1:**
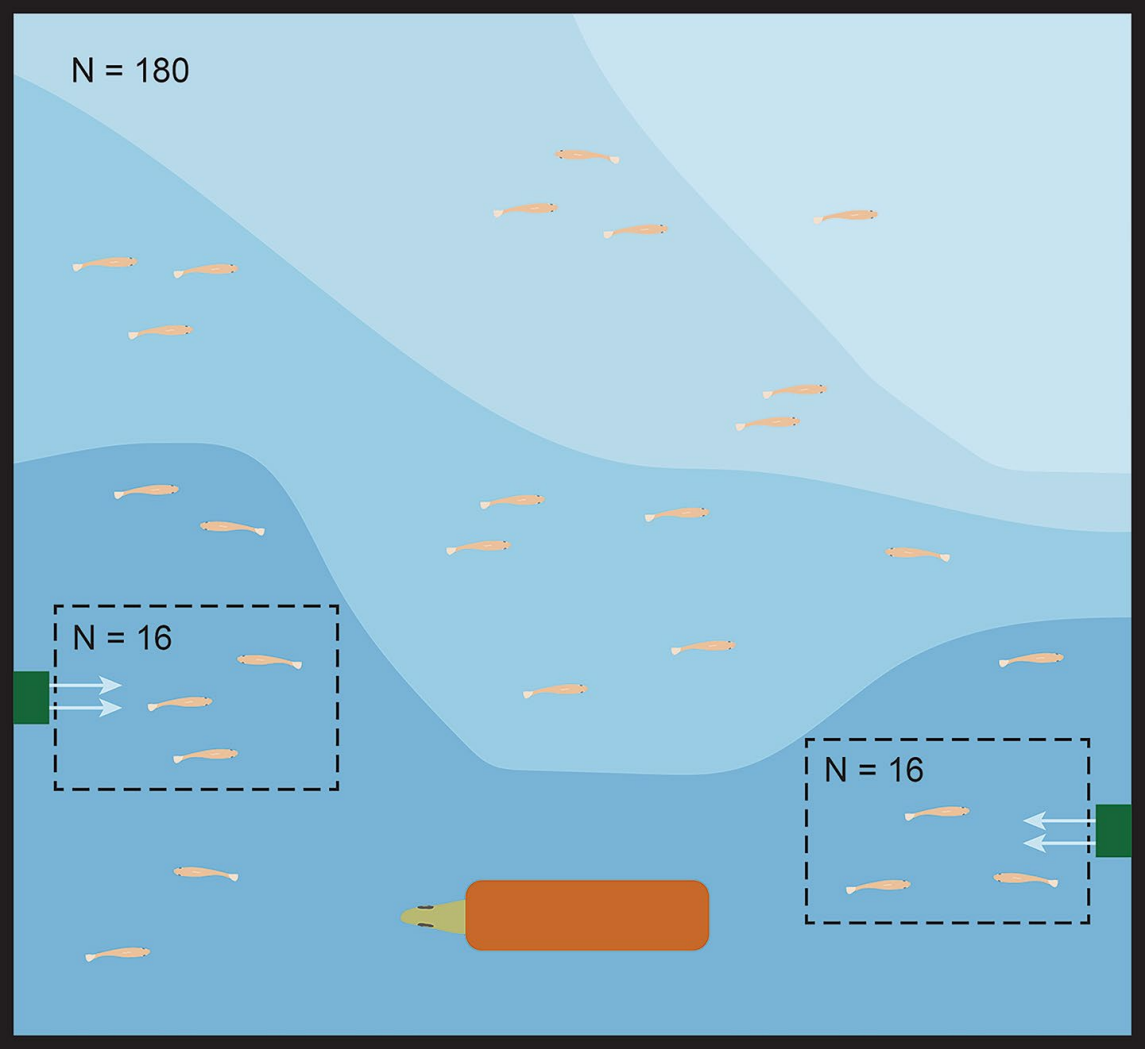
Set up of the experimental tank (view from above). Fish from the predation treatment were allowed to swim freely in the tank (N = 180), whereas control fish were placed in 11 L transparent tanks (N = 16 each, shown in dashed squares) to provide visual cues, with a filter pump that allowed water to get into the tank to provide olfactory cues. Water flow from the pumps is indicated with arrows. A predatory cichlid was placed at the deepest area with a clay pipe for shelter. Different shades of blue represent different depths

The experimental tank was 120⋅110⋅70 cm, filled with 220 L of water, with a bottom layer of multicoloured limestone gravel (3–8 mm grain size) with which we crafted areas of different depths, so that the water depth ranged from 5 to 17 cm (Fig. 1). The shallow area provided a refuge for the guppies where the predator could not hunt. One cichlid (*Crenicichla alta*) was placed at the deepest area of the tank and provided a clay pipe as a shelter (Fig. 1). The cichlids used were acquired through the aquarium trade and fed with live guppies prior to the experiment. Note that this is a sister species to *C. frenata*, the guppy’s natural predator (Varella et al. 2018). Control fish (16 individuals per tank) were held in two 11 L transparent tanks which were located at each side of the experimental tank. We installed two Eheim filter pumps (60 L ⋅ h^− 1^ per pump) outside the 11 L tanks and directed the water flow into each of these 11 L tanks to provide olfactory cues for the control fish. Thus, control fish had visual exposure to the cichlid, to the behaviour and density of guppies in the full tank, and were exposed to the same water. This setup had the potential limitation that control fish were in a more constrained area, which may affect their growth and development. However, we prioritised standardising the visual and olfactory cues of predation which are known to have strong developmental effects on guppies (Torres-Dowdall et al. 2012; Ghalambor et al. 2015), including on their relative brain size (Reddon et al. 2018). Thus, we effectively controlled for these effects to better focus on selective survival.

During the first two days of the experiment, we placed a mesh enclosure around the cichlid so guppies could escape easily, acclimatise, and learn about the position and potential danger of the predator. This mesh was removed on the third day. We visually monitored the number of fish in the tanks daily. When the number of surviving fish seemed to have reached our target (15% of the group, ∼ 30 individuals), they were captured with a net to be counted. If more than the desired number of survivors was counted, they were returned to the tank. Due to logistical constraints, the final percentage of survivors varied from 13 to 23% between replicates. The number of weeks fish were in the treatment varied from 3 to 7 weeks for males, and from 11 to 14 weeks for females. See Supplementary material Sect. 1 for details. Because one predator showed signs of stress (hiding and very little feeding), it was replaced with another cichlid after 28 days. Fish were kept at 24 °C under a 12:12 light:dark cycle and fed flake food daily and freshly hatched brine shrimp at least three times per week.

### Body size and brain measurements

To test for differences in body size between treatments, photographs of fish were taken before and after the predation event by placing 30 fish at a time in a 4 L tank with 2 cm of water and photographed from above with a Nikon DSLR camera. Images were then measured using Image J software (Abramoff et al. 2004) to obtain individual’s standard length (from the tip of the snout to the end of the caudal peduncle).

To test for differences in relative brain size and relative brain regions, 12 individuals from each sex, replicate, and treatment group (N = 144) were randomly selected and their standard length was measured to the nearest 0.01 mm using a digital calliper. Fish were then placed under a dissection microscope (Leica MZFLIII) and brains dissected and stored in PBS. To quantify brain region volumes, brains were photographed from the dorsal, ventral, left, and right side under the dissection microscope with an attached digital camera (Leica DFC 490) and then weighed to the nearest 0.01 mg (VWR SM-425i-C precision scale). The length, width, and height of the olfactory bulbs, telencephalon, optic tectum, hypothalamus, cerebellum, and dorsal medulla were then measured using Image J following (Kotrschal et al. 2012). See Supplementary material Sect. 3.2.1 for details. The volume of each of the brain regions was estimated using: $V =(L \times W \times H)\frac{\pi }{6}$. All body size and brain measurements were taken blind to treatment.

#### Statistical analyses

To test whether body size before and after the predation event differed between treatments we calculated the standardised mean difference for each group (SMD, Hedges and Olkin 1985), which is the difference in body size between the time fish started the experiment and when they finished the experiment. Note that fish were not individually marked, and that sample sizes were the same in the control treatment before and after the experiment but different in the predation treatment as at the end of the experiment we could only measure survivors. SMDs for each observation with their associated variances were used as dependant variables in a random meta-regression model using the *metaphor* package (Viechtbauer 2010). We included treatment and replicate as fixed effects, and observation ID as a random effect. We ran these models separately for males and females as sex differences in guppies are considerable.

To test for differences in relative brain size and relative brain regions between treatments, we log-transformed body size (mm), brain weight (mg), and brain region volume (mm^3^) before the analyses. These analyses were fitted separately for males and females as treatment duration (the time in the predator tank) varied between the sexes (estimate males = -51, se = 10.970, F = 21.615, p = 0.009), and the highly pronounced sexual dimorphism makes sex comparisons not too meaningful.

To test for differences in relative brain size, we ran a linear model for brain weight, with the predictors of treatment, body size, replicate, and all two-way interactions. Log-body size was fitted as a covariate to account for allometry and focus on treatment differences in *relative* brain size. We checked whether replicate interacted with treatments or allometries, but as these were not parameters of biological interest, we removed non-significant interactions from the model (all p > 0.2).

To test for an effect of predation treatment on relative brain region volumes, we fitted a multivariate linear model for each sex with the predictors of treatment, brain weight, replicate, and all two-way interactions. All interactions in this model were non-significant and uninformative for our research question and therefore removed (all p > 0.9). We also ran univariate models for each brain region (details of those models and results are available in the Supplementary Material). All statistical analyses were performed in R v.3.6.1 (R Development Core Team 2012) and model terms were tested for significance using the ANOVA function in the *car* package (Fox and Weisberg 2011) specifying Type III Wald chi-square tests. The model results and code are available in the Supplementary Material—https://osf.io/42cpt/.

## Results

### Effects on body size

**Fig. 2:**
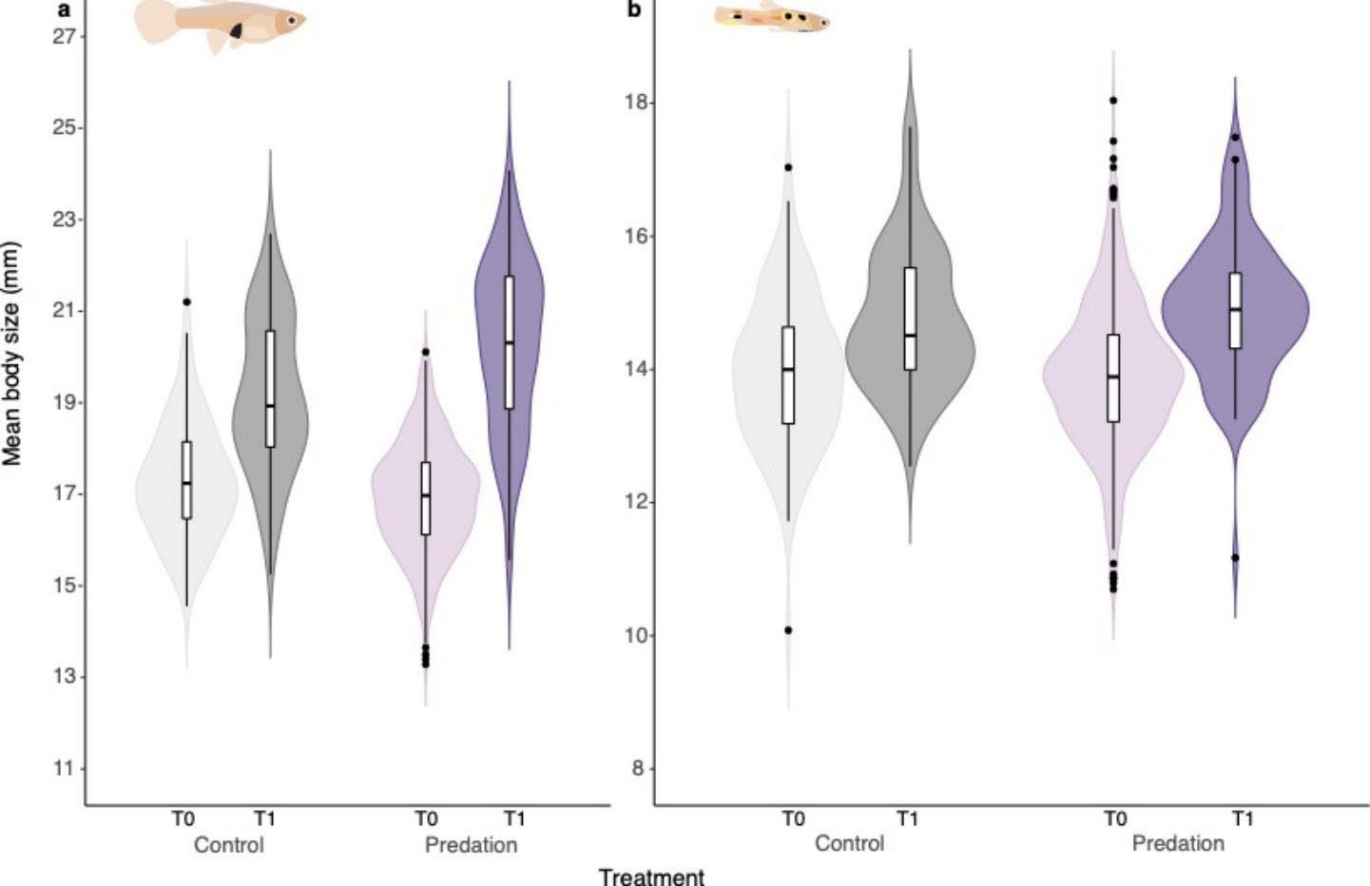
Body size for (a) females and (b) males for control (grey) and predation (purple) treatment fish measured before (T0; light colours) and after (T1; dark colours) the predation event. The white box indicates the median and interquartile range (IQR), whiskers extend to 1.5*IQR. Black data points indicate values outside the 1.5*IQR. The violin shapes show the distribution of the data

We found sex-dependent effects of predation on body size. In females, body size differed between treatments, with surviving females being larger than control females (estimate = 1.322, s.e. = 0.505, Z = 2.619 p = 0.008; Fig. 2a; Suppl. 2.2.2 & 2.2.3). The magnitude of this difference in body size varied between replicates (estimate replicate 2 = 1.094, s.e. = 0.602, Z = 1.817, p = 0.069; estimate replicate 3 = 2.673, s.e. = 0.625, Z = 4.277, p < 0.001).

In males, body size between survivors and controls did not differ (estimate = 0.303, s.e. = 0.187, Z = 1.622, p = 0.105; Fig. 2b; Suppl. 2.2.2 & 2.2.4), nor was it affected by replicate (Suppl. 2.2.4).

### Effects on relative brain size

**Fig. 3:**
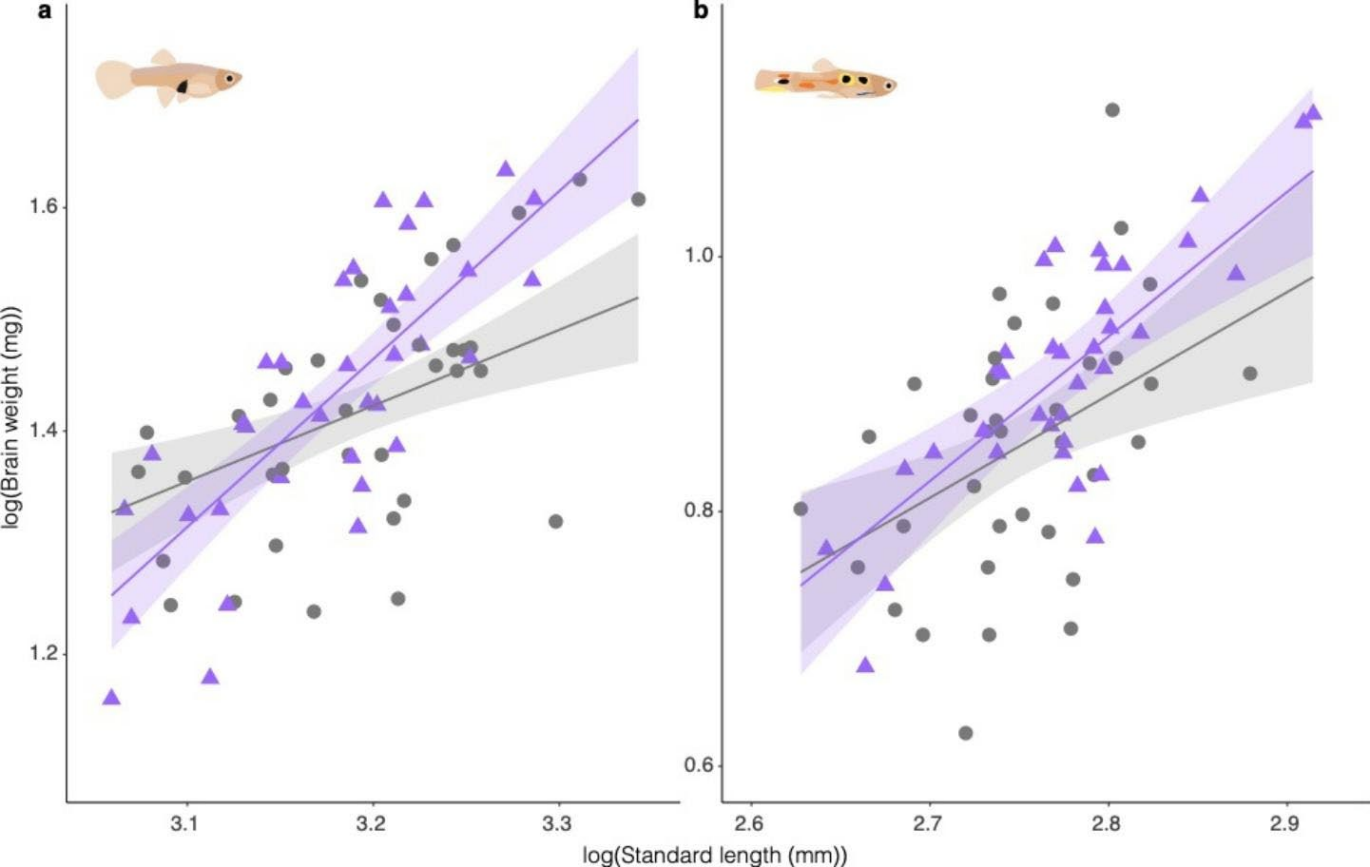
Differences in brain size between the control (grey circles) and predation (purple triangles) treatments for (a) females and (b) males. Model predictions are plotted as the best fit line with 95% CI

In females, we found that predation impacted the allometry between brain weight and body size, with surviving females having relatively larger brains than controls at large body sizes and relatively smaller brains at smaller body sizes (treatment ⋅ body size estimate = 0.825, s.e. = 0.260, F_1,66_= 19.051, p = 0.002; Fig. 3a). Note that at smaller to intermediate sizes, the difference between surviving females and controls was not as large (Fig. 3a). These results varied between replicates (F_2,66_ = 17.378, p < 0.001). In males, we found no difference in the allometry between brain weight and body size between treatment groups (treatment ⋅ body size estimate = 0.328, s.e. = 0.327, F_1,66_= 1.006, p = 0.319; Fig. 3b). Additionally, overall relative brain size was similar between treatments (treatment estimate = − 0.871, s.e. = 0.902, F_1,66_= 0.933, p = 0.338; Suppl. Section 3). We observed a body size effect on relative brain independent of treatment; larger males had relative bigger brains (body size estimate = 0.807, s.e. = 0.239, F_1,66_= 11.429, p = 0.001; Suppl. Section 3). These results varied between replicates (F_2,66_ = 3.672, p = 0.031), but note there was no replicate by treatment interaction.

### Effects on relative brain region size

The multivariate analyses of brain region volumes (olfactory bulb, telencephalon, optic tectum, hypothalamus, cerebellum, and dorsal medulla) for females and for males after accounting for the allometries associated with brain weight (females: F_6,62_ = 25.030, p < 0.001; males: F_6,62_ = 13.449, p < 0.001) revealed no effect of treatment on relative brain region volumes for either sex (females: F_6,62_ = 0.479, p = 0.821; males: F_6,62_ = 1.130, p = 0.356). Univariate models of each brain region supported the lack of differences and are available at the Suppl. Section 3.2.

## Discussion

To test if predators can exert a direct effect on the brain by selectively removing prey with specific brain traits, we compared guppies that survived a predation event (survivors) to guppies that were exposed to olfactory and visual predator cues only (controls). We found that survivor females were bigger and showed a body-size-dependent difference in brain size compared to controls, while no such differences were observed in males. In both sexes, relative brain region volumes were similar between survivors and controls. Our study therefore provides experimental evidence that direct predation can impact the relative brain size of prey.

### Values and constraints of our experimental approach

To our knowledge, only one previous study has experimentally assessed the selection effect of predators on prey brain anatomy under semi-natural conditions (Samuk et al. 2018). The added strength of our study is that we tested for a selective effect of predators on prey brain while controlling for plastic changes from predator cues and for environmental confounding factors such as food availability. The value of our control treatment is that we aimed to control for plasticity induced by a predator as it is well known that either direct exposure to predators or its cues, even for short periods of time during adulthood, can cause plastic changes (Gonda et al. 2012; Lucon-Xiccato et al. 2020).

We assessed brain morphology of adult guppies that had to survive a predator *versus* control fish that were exposed to the same predator but could not be eaten, in an effort to control for plastic changes due to predation risk cues and specifically test for an effect of selection by the predator. However, given the nature of the experiment, several weeks passed between the predation event and the assessment of body size and brain morphology, and so we cannot fully disentangle the effects of natural selection and phenotypic plasticity. Did our results arise from non-random predation of females with certain brain and body sizes, or did the period of exposure to the predator trigger plastic changes in the brain during the weeks following exposure? The literature provides evidence for both those mechanisms. For instance, life history traits, growth, and brain anatomy have all been shown to respond rapidly to natural selection (Roff 1992; Stearns 1992; de Winter and Oxnard 2001), and adult guppies show plasticity in all three traits following early life exposure (Reznick 1990; Reznick and Yang 1993; Burns and Rodd 2008). More importantly, plasticity and natural selection may not necessarily work in the same direction. For instance, plasticity could lead to lower somatic growth on body size, while natural selection could increase somatic growth (Handelsman et al. 2013). Evidence suggests however, at least for brain size in guppies, that both plasticity and natural selection seem to drive responses in the same direction when exposure to predators in early ontogeny occurs (Reddon et al. 2018). While we could not completely disentangle evolutionary from plastic effects, our results reveal that an episode of predation during adulthood can influence brain size, independently of other ecological confounding factors present in the wild. To further dissociate predation-driven effects due to direct selection for specific brain traits from phenotypic plasticity, and therefore further our understanding of how predators are able to cause changes in the brain, future work should assess brain and body size of controls and survivors immediately after exposure to the treatment, as well as include a treatment where control fish are held in tanks of similar size as those of the predation treatment.

### Body-size-dependent difference in brain size in females, but not males

The brains of female guppies were affected by the predation treatment. This resembles results from a previous study where a large-scale survival experiment under semi-natural conditions showed that relative brain size determined survival in females, but not in males (Kotrschal et al. 2015). However, the results we report here deviate from this previous study and from our predictions as we did not find a clear effect of predation on relative brain size at all size classes. Instead, predation changed the slope of the regression between female brain and body size, which resulted in relatively smaller brains in small survivors but relatively larger brains in large survivors, compared to controls. This was unexpected but interesting, as it suggests that brain-size derived cognitive advantages (e.g. Kotrschal et al. 2013; Benson-Amram et al. 2016; Buechel et al. 2016) may be size-dependent under predation pressure. Relatively larger brains might provide cognitive advantages if indeed a relatively larger brain helps to avoid getting eaten (Moller and Erritzoe 2014), but may also be costly to maintain. Thus, targeting of size-classes by predators (e.g. Johansson et al. 2004), or different escape strategies used by small and large fish may be causing the allometric effects we found. Such body size-dependent effect of predation on brain size has been shown in male killifish when comparing high predation sites versus sites with no predators (Dunlap et al. 2019), but seems absent in other studies relating predation pressure to brain size (Walsh et al. 2016; Reddon et al. 2018; Mitchell et al. 2020). This may be due to a mix of differently sized predators in the wild, either across predatory species or from changes with age/size of gape-size limited predators like *Crenicichla*. In males, there was no difference in relative brain size. This could be partially attributed to the fact that male body growth is determinate (see below) constraining variation in body size. Indeed, in small females (body size comparable to male range) there was no difference in relative brain size. It is also likely that differential predation may be masked by male’s conspicuous body colouration Houde 1997; Kotrschal et al. 2015; but see Reddon et al. 2018).

### No differences in brain anatomy between survivors and controls

In addition to overall brain size, variation in specific brain regions may play a fundamental role in how animals respond to their environment, and indeed changes in specific brain regions can be associated with predation risk (Joyce and Brown 2020). Despite predicting differences between survivors and controls on specific brain regions such as the telencephalon and optic tectum as they are associated with cognitive and motor functions (Broglio et al. 2003; Triki et al. 2021), we found no such effect. Previous studies have shown plastic changes in specific brain regions in response to predation and other ecological pressures (Gonda et al. 2013; Samuk et al. 2018; Joyce and Brown 2020). Similarly, predator cues have been linked with neurogenesis and transcriptomic changes in the brain (Sanogo et al. 2011; Dunlap et al. 2017). Future work looking at specific cell proliferation or molecular changes in a multi-generational selection experiment should provide a fruitful avenue to further disentangle the effects of predation on the brain.

### Body size differences between survivors and controls

The body size difference in survivor and control females that we found could be explained by size-selective predation if the pike cichlid preferentially preyed on smaller females. In the wild, high predation guppies are typically smaller in body size than low predation fish (Reznick et al. 2001; Reddon et al. 2018), but this effect is likely due to the strong selection for maturing early and at smaller size in risky habitats rather than due to size-selective predation (Reznick 1990; Reznick et al. 1996). Differences in body size associated with predation have indeed generated conflicting results. For instance, males exposed to predation risk cues in the laboratory during development were bigger than those exposed to control cues, but female body size showed no response (Reddon et al. 2018). In another study, a comparison of a laboratory-born generation of guppies from high and low predation localities showed that females from high-predation sites grew faster than those from low-predation sites (Arendt and Reznick 2005). While *Crenicichla* cichlids often prefer larger prey (Johansson et al. 2004), predation across prey sizes by cichlids, rather than selective predation on large guppies, has also been described (Mattingly and Butler 1994). Moreover, other predators present in wild populations target smaller size classes (Rodd and Reznick 1997). In our case, smaller females may have been easier to catch than larger ones by the cichlid, since body size is a key factor influencing swimming parameters (Rubio-Gracia et al. 2020), and in our study females were virgin and so their swimming performance was not compromised by pregnancy (Banet et al. 2016). This hypothesis may also explain why in our study males were predated faster than females, as cichlids would have required more (small) males than (large) females to reach satiation (as seen by Mattingly and Butler 1994).

The lack of body size differences in males between the treatments may be explained by the fact that male guppies show almost determinate growth with little additional growth after maturation, while females continue to grow substantially during adulthood (Constantz 1989; Arendt and Reznick 2005). Due to the large number of animals necessary for the project, breeding all animals took several months. This means that, while all focal animals were adults, they were between four and seven months old. This may have produced a larger range in female compared to male body size and hence a stronger potential to detect size-selective mortality. Alternatively, in a scenario relying on phenotypic plasticity, survivor females may have simply grown faster during the time in the predation treatment tank. This could be due to the fact that controls and survivors, although designed to only differ in the potential for physical contact between guppies and pike cichlid, also differed in the space they could utilize. Controls were restricted to smaller tanks within the predator tanks whereas survivors could use the larger tank. As growth in fish can depend on tank size (Espmark et al. 2017), this may have contributed to our results. While the lacking body size difference in males may indicate such a scenario is unlikely, the near-determinate male growth explained above may render this counter-argument invalid. It is hence evident that dedicated growth experiments in the set up used here, but without a predator, are needed to conclusively clarify the mechanism by which females that survive predation are larger than controls.

## Conclusions

Predation can lead to differences in absolute and/or relative brain size across species (Moller and Erritzoe 2014), and even among populations of the same species (Burns and Rodd 2008; Walsh et al. 2016; Kotrschal et al. 2017). Here we show that direct predation in adulthood impacts relative brain size and uncover a sex- and body-size dependent effect. Our results highlight the need to explore the complex effect of predation on brain evolution further, and ultimately incorporate cognitive assays to understand whether individuals evolve larger brains and better learning capacities to avoid predators.

## Data Availability

Data and code are available at https://osf.io/42cpt/.
